# The Treatment Outcomes of Tuberculosis Patients at Adare General Hospital, Southern Ethiopia (A Five-Year Retrospective Study)

**DOI:** 10.3390/tropicalmed9110262

**Published:** 2024-11-02

**Authors:** Bizunesh Tsegaye, Zufan Bedewi, Solomon Asnake

**Affiliations:** 1 Sidama Region Education Office, Hawassa, Ethiopia; bizuneshtsegaye6@gmail.com; 2 College of Natural and Computational Sciences, Hawassa University, Hawassa P.O. Box 05, Ethiopia; zufanw2006@yahoo.com; 3 College of Medicine and Health Sciences, Hawassa University, Hawassa P.O. Box 1560, Ethiopia

**Keywords:** TB treatment outcome, tuberculosis, DOTS, treatment success rate

## Abstract

Ethiopia is among the countries most heavily affected by tuberculosis, where it is the leading cause of morbidity, the third cause of hospital admission and the second cause of death. To improve tuberculosis management and control, the early detection of cases, effective treatment and the persistent evaluation of treatment outcomes are vital issues that should be taken into consideration. This study was designed to determine the treatment outcomes and associated risk factors among TB patients registered at Adare General Hospital in Southern Ethiopia. A five-year retrospective study was conducted by reviewing the files of the TB patients registered from September 2013 to August 2017. The data were coded, cleaned, entered and analyzed using SPSS version 20 statistical software. Bivariate and multivariable logistic regression with odds ratios (OR) along with a 95% confidence interval was computed and interpreted. A *p*-value < 0.05 was declared as statistically significant. Among the 1122 cases, 620 (55.3%) were male, 748 (66.7%) were from urban areas, 319 (28.4%) were smear positive, 352 (31.4%) were smear negative and 451 (40.2%) were extra-pulmonary patients. Among the treated patients, 284 were declared cured, 753 completed their treatment, 29 were defaulters, 3 failed to follow up, and 53 died. The overall treatment success rate was 92.4%. The TB patients from urban areas (AOR = 1.44, 95% CI: 0.28, 0.67), and the HIV-negative TB patients (AOR = 5.48, 95% CI; 3.474, 8.64) were significantly associated with successful treatment outcomes. The treatment success rates of tuberculosis at Adare General Hospital were comparable to the national health facility-level coverage, but they should be maintained and strengthened further to attain tuberculosis-related national and millennium goals.

## 1. Introduction

Tuberculosis (TB) is a communicable disease that is caused by *Mycobacterium Tuberculosis* and that mainly affects the lungs, can cause pulmonary tuberculosis of other organs, and can cause extra-pulmonary tuberculosis [1]. It is a major public health problem in the world, being among the ten top leading diseases that cause death [2]. According to a WHO report, in 2017, about 10 million people were infected and 1.6 million died due to TB [3]. About 56% of the cases were from Southeast Asia and Western Pacific Regions, and 29% cases were from Africa, where the highest rate of death occurs [4].

Among the 30 highest TB-burdened countries in the world, Ethiopia ranks seventh, and the estimated TB prevalence rate in this country is 27/1,000,000 of the population, with an estimated incidence rate of 224 per 100,000 people [3]. Annually, there are an estimated 5000 or more Multi-Drug Resistance (MDR–TB) cases; hence, according to the report from the WHO, among the 27 highest MDR-TB-burned countries, Ethiopia was placed at a rank of 15 [5]. The health minister of the country indicated that this disease was the leading cause of morbidity, the third leading cause of hospital admission, and the second cause of death [6]. The globally accepted TB intervention strategy is Directly Observed Treatment Short-Course (DOTS). In 1992, the DOTS strategy was started as a pilot project in Ethiopia and then scaled up and implemented at the national level [6]. Then, all the public, private and non-governmental health facilities provided the service [7]. As various studies conducted in different health institutions in the country indicated, the treatment outcomes showed incremental progress after the delivery of DOTS services [8,9,10,11,12], though other studies indicated the presence of various challenges [13,14]. Adare General Hospital implemented a DOTS strategy starting in the national scale-up period; however, the institutional status of the treatment outcomes and associated challenges have not been studied so far. Therefore, this retrospective study aimed to assess the treatment outcomes of TB patients and identify the factors associated with unsuccessful outcomes in Adare General Hospital in Southern Ethiopia.

## 2. Methods

### 2.1. Description of the Study Area

**Figure 1:**
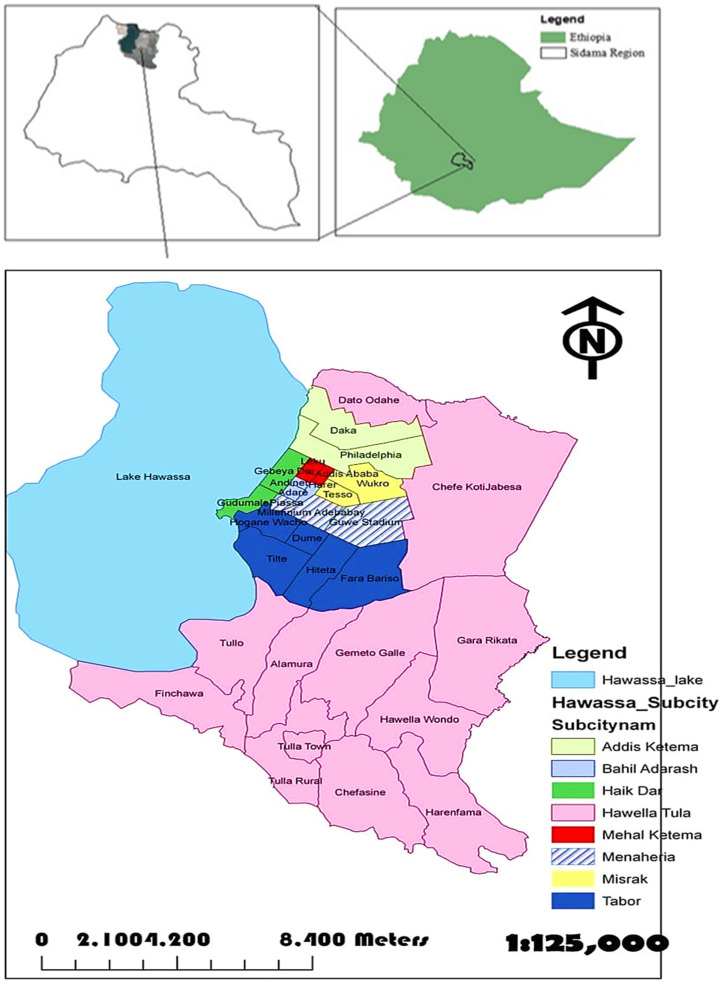
Map of the study area (Source: Hawassa City Administration office).

Hawassa, the capital city of the Southern Nations, Nationalities, and Peoples‘ Region (SNNPRS) is located about 275 km south of Addis Ababa. Geographically, it lies at a 7°5′ latitude N and 38°29′ longitude E, at an altitude of 1708 MASL (meter above sea level). The mean annual rainfall and temperature of the area varies from 800 to 1000 mm and 20.1 °C–25 °C, respectively (Figure 1). Based on the 2007 National Population and Housing Census, Hawassa has 329,734 inhabitants of which 169,677 are male and 160,057 are female. Adare General Hospital is located at the center of the city and has 70 functional beds. The hospital has one DOTS clinic that performs in accordance with the National TB and Leprosy Control Program (NTLCP) guidelines of Ethiopia. The clinic provides basic treatment and diagnostic services for all forms of TB through clinical examination, the Ziehl–Nielsen staining method, or sputum smears examination and chest radiographs. Patients diagnosed with TB were registered and treated according to the NTLCP guidelines [6].

### 2.2. Study Design, Period and Data Collection

An institutional-based retrospective study was conducted from April to May 2018 via reviewing the registration book of tuberculosis patients registered and treated from 2013 to 2017 at the DOTS clinic of Adare General Hospital. Registered data with complete information were collected retrospectively by trained health professionals using a structured checklist and used as a source of data for the investigation. The basic required information such as the patients’ sex, age, address, date of treatment started, date of treatment completed, HIV sero-status, type of TB case, and treatment outcome.

### 2.3. Statistical Analysis

The retrospective data were entered into a Microsoft Excel sheet and cleaned, then transported to the SPSS version 20 statistical software. The frequency and percentage of the data were determined using descriptive statistics. A logistic regression statistical model was used to assess the relationship between the dependent and independent variables. A bivariate logistic regression model was used to evaluate the pairwise association between the variables, and the variables with a *p* value < 0.25 at a 95% confidence interval were considered to be significant. The variables with significant association were entered into a multivariate logistic model and computed to determine the potential independent predictor variables of TB treatment outcomes at a 95% confidence interval. Adjusted odds ratios (AOR) with a 95% confidence interval (CI) were used to determine the strength of the association of the variables, and a *p*-value < 0.05 was considered as statistical significance.

### 2.4. Ethical Considerations

Institutional ethical clearance was obtained from the ethical Review Committee of Hawassa University (Ref. No. HUVPRTT/9182/2022). A supportive letter was obtained from Hawassa University and the Adare General Hospital medical director’s office approved the utilization of the retrospective data. The permission to adopt a map of Hawassa was obtained from the Hawassa City Administration.

### 2.5. Operational Definition

The following clinical case and treatment outcome operational terms were used in this article based on the standard definitions the NLCP adopted from the WHO [6]:

Smear-positive pulmonary TB (PTB+): A patient with at least two sputum specimens that were positive for acid fast bacilli (AFB) by microscopy, or a patient with only one sputum specimen that was positive for AFB by microscopy, and chest radiographic abnormalities consistent with active PTB;

Smear-negative pulmonary TB (PTB−): A patient with symptoms suggestive of TB, with at least two sputum specimens that were negative for AFB via microscopy, and with chest radiographic abnormalities consistent with active PTB, or a patient with two sets of at least two sputum specimens taken at least two weeks apart, which were negative for AFB via microscopy, and radiographic abnormalities consistent with PTB, and a lack of clinical response to one week of broad spectrum antibiotic therapy;

Extra-pulmonary TB (EPTB): This included the TB of organs other than the lungs, such as lymph nodes, the abdomen, the genitourinary tract, the skin, joints and bones, the meninges and others. The diagnosis of EPTB was based on fine needle aspiration cytology or biochemical analyses of cerebrospinal/pleural/ascitic fluid or the histopathological examination or strong clinical evidence consistent with active EPTB, followed by the decision of a clinician to treat with a full course of anti-TB chemotherapy. In all the cases of EPTB, the sputum examinations and chest radiographs were used to rule out the involvement of lung parenchyma. This hospital lacks the facilities for culture and drug susceptibility testing.

Categories of treatment outcomes:

Successful outcome: if the TB patients were cured (negative smear microscopy at the end of treatment and on at least one previous follow-up test) or completed treatment with the resolution of their symptoms;

Unsuccessful outcome: if the treatment resulted in treatment failure (remaining smear positive after 5 months of treatment), defaulted (patients who interrupted their treatment for 2 consecutive months or more after registration), or died.

## 3. Result

### 3.1. Socio-Demographic and Clinical Characteristics of the Patients

A total of 1151 tuberculosis patients were registered in the TB registrar unit of the hospital, of which 1122 (97.5%) tuberculosis cases had complete records on their treatment outcome. Among the total registered TB patients with complete records (*n* = 1122), 620 (55.3%) were male and 66.7% (*n* = 748) of the patients were urban residents. The majority (61.5%) of the TB cases were between 15 and 34 years of age, and children below 14 years accounted for 9%. Smear positive (PTB+), smear negative (PTB−) and extra-pulmonary TB (EPTB) cases were 319 (28.4%), 352 (31.4%) and 451 (40.2%), respectively. Most of the TB patients were new (1064 (94.8%)), while relapse, transfer-in and treatment after failure accounted for 48 (4.3%), 8 (0.7%) and 2 (0.2%), respectively. All of the registered cases for anti-TB treatment were tested for HIV voluntarily and 238 (21.2%) of the study participants were HIV-positive (Table 1).

### 3.2. Treatment Outcome of the Participants

The treatment outcomes of most of the study participants were successful; 753 (67.1%) completed their anti-TB treatment and 284 (25.3%) were cured. Treatment failure, death and defaulting accounted for 3 (0.3%), 53 (4.7%), and 29 (2.6%) cases, respectively. We excluded 29 TB patients (transferred-out cases) from the treatment success rate analysis, since the status of the patients were not known. The majority of treatment failure and defaults were observed in the ages of 45–54 and 55–64, respectively, and the highest rates of deaths (15%) were registered among HIV/TB co-infected patients (Table 2).

### 3.3. Treatment Outcomes of TB and Their Trends

We analyzed the treatment outcomes of 1122 tuberculosis patients who were registered at the hospital during the study period from January 2013 to December 2017. The death, default and failure rates of the tuberculosis patients showed incremental progress from 2013 to 2017, though the death rate seemed stagnant in 2013 and 2014. In general, the unsuccessful (poor treatment outcome) rate increased from 2014 to 2017 (3 to 12.8%). The cure rate of the TB patients increased year to year from “2013 to 2017”, though it decreased in 2015. The treatment completion rate of the patients decreased from “2013 to 2017”. The success rate of the TB patients decreased from “2013 to 2017”, though the overall five-year treatment success rate of the TB patients was 1037 (92.4%) (Table 3).

### 3.4. Treatment Success Rate and Its Associated Factors

The treatment success rate was 51% in males and 41.4% in females. The majority (63%) of the patients with a successful (favorable) outcome were urban residents and 29.4% were rural residents. The treatment success rates were 37.3%, 29.2% and 25.9% among EPTB, PTB− and PTB+ patients, respectively. The success rates among the new and relapse cases were 88.6% and 3.8%, respectively. In addition, a high treatment success rate was observed among the TB patients of age 0–14 and 45–54 years.

A logistic regression analysis was performed to identify the independent predictors of treatment outcomes among TB patients (Table 4). In the case of bivariate analyses, the following explanatory variables, were significantly associated with the treatment outcome (*p* < 0.25): place of residence (urban or rural), HIV status (positive or negative) and types of TB (PTB+, PTB− or EPTB). These variables were entered into a multiple logistic regression model to find the potential predictor. After computing, we found that living in an urban area and being HIV negative was significantly associated with a successful treatment outcome (*p* < 0.05). Accordingly, as the adjusted odds ratio (AOR) result indicated, TB patients from urban areas were 1.44 times (AOR = 1.44, 95% CI: 0.28, 0.67) more likely to achieve a successful treatment outcome compared to cases from rural areas. Regarding HIV status, HIV-negative TB patients were 5.48 times (AOR = 5.48, 95% CI; 3.474, 8.64) more likely to achieve a successful treatment outcome as compared to HIV-positive patients. Though there was no significant association between types of TB cases and treatment outcomes, pulmonary tuberculosis-negative (PTB−) cases were 0.95 times (AOR = 0.95, 95% CI; 0.26, 1.86) more likely to achieve successful treatment outcomes as compared to pulmonary tuberculosis-positive (PTB+) cases. Moreover, patients with extra-pulmonary TB (EPTB) cases were 0.70 times (AOR = 0.70, 95% CI; 0.26, 1.86) more likely to achieve successful treatment outcomes as compared to patients with pulmonary tuberculosis positive (PTB+) cases (Table 4).

## 4. Discussion

In this study, males accounted for more than half of the study participants; correspondingly, the studies conducted in different health institutions in the country also indicated that more males were involved [15,16]. However, findings from studies conducted by Gambella [17] and Raya Kobo [18] contrasted with this. Most (83.4%) of the study participants were 15–54 years old; the WHO also reported that the productive age was more affected by tuberculosis and associated morbidity and mortality [19]. This situation might have a negative impact on the economic and social development of the community and the nation at large. In the present study, the majority (671 (59.8%)) were pulmonary TB patients, and 451 (40.2%) were extra-pulmonary TB patients; as to the TB profile data, high proportions of TB patients presented cases of EPTB and smear-negative PTB, which is comparable to other TB profile data in the country [15,20,21,22,23].

The treatment success rate was 92.4%, which is lower than the success rate (94.8%) reports of northwest Ethiopia [22]. However, the presented success rate was higher than those of other reports in different parts of Ethiopia such as, Tigray (89.2%) [21], Gambella (86.1%) [17], Dilla (85.3%) [15], Nekemte (70.8%) [24] and Hossana (43.3%) [25]. The unsuccessful TB treatment outcome rate was 7.6%; this finding is lower than the unsuccessful treatment outcome reported in Dilla (14.7%) [15], Gambella (13.9%) [17] and Tigray (10.8%) [21]. This could be associated with differences in socio-demographic characteristics of the patients, the appropriateness of the institutional setup, the follow-up and counseling of the patients in DOTS clinics, and the knowledge and attitude towards side effects due to DOTS [26].

The death rate (4.7%) was lower than that reported from Ethiopia (5%) [18], Nigeria (6.5%) [27] and Zimbabwe (8.7%) [28]. However, this finding was higher than the death rate reported from Ethiopia (3% and 3.4%) [15,25], Turkey (2.4%) [29] and Brazil (2.8%) [30]. The default rate in this study was (2.6%), which might be due to better supervision and health education activities. This finding was lower than the studies conducted in Ethiopia (3.2%, 8% and 11.1%) [15,21,31], Uzbekistan (6%) [32], Turkey (3.9%) [29] and Nigeria (9.8%) [27].

The treatment failure rate of 0.3% was inconsistent with the rate reported in another study in Ethiopia (0.3%) [15], and higher than the treatment failure rate in Malaysia (0.2%) [33]. Nonetheless, the rate observed in this study was lower than that of the findings from Ethiopia (0.5, 2.2%, and 3.7%) [18,21,25], Uzbekistan (3%) [32], Turkey (1.1%) [29], Brazil (2.1%) [30] and Nigeria (1.5%) [27]. The overall TB-HIV co-infection rate was 21.2%, which was lower than that from the studies conducted in Gonder, northwest Ethiopia (52.1%) [10] and the Gambella region (26.12%) [17]. However, it was higher than (12.7%) Debre Tabor, Northwest Ethiopia [34], (16.5%) Hossana, Southern Ethiopia [25], (11.7%) Gojam, northwest Ethiopia [22] and (17.1%) Nekemte, Western Ethiopia [24].

The present study indicated the presence of an association between treatment outcome and HIV status; hence, as compared to HIV-positive TB patients, HIV-negative TB patients were five times more likely to achieve successful treatment outcomes. This finding was in line with investigations conducted in Ethiopia [21,35], Somalia [36], South Africa [37], Finland [38] and Brazil [30], where HIV-negative TB cases had a higher likelihood of treatment success as compared to TB/HIV co-infected cases. In the case of TB/HIV co-infected patients, due to the fact that HIV infection declines CD4 cell counts progressively by about 50–80 cells/mm^3^ per year, the immune status of the individual might not be efficient enough to prevent the dissemination of *Mycobacterium tuberculosis*, which might decrease treatment success [39]. Also, it might be associated with the co-administration of ART along with anti-TB therapy, which can lead to drug–drug interactions, overlapping drug toxicities and immune reconstruction syndrome [40].

Residence and treatment outcomes were also highly associated; patients from urban settlement were 1.5 times more likely to have successful treatment outcomes as compared to patients from rural areas. This result was consistent with studies conducted in Southern Ethiopia [15,41] and Central Ethiopia [42], but contrary to studies conducted in Southern Ethiopia [43]. As suggested by [41], the better treatment outcomes in urban areas could be due to higher awareness about treatment procedures as compared to rural residents’, whose treatment outcome successes were lesser due to the lower awareness of TB treatment and the long distance between their homes and the treatment centers. Though there was no significant association between treatment outcomes and TB type, pulmonary TB (PTB−) cases were 0.95 and 0.7 times more likely to have successful treatment outcomes as compared to pulmonary TB (PTB+) and extra-pulmonary TB (EPTB) cases. This finding was in line with the study conducted in Hossana, South Ethiopia [25], but contrary to the finding of the study conducted in northwest Ethiopia [22].

## 5. Conclusions

The five-year retrospective study indicated that the DOTS strategy improved TB treatment success in Adare General Hospital. The treatment outcome rates of the registered patients in the study area were high and met the target success rate set by the WHO. Successful treatment outcomes were aligned to the urban community as compared to rural residents and HIV-negative cases, and compared to the positive ones. Hence, awareness creation through health education for rural patients and the regular follow-up of patients with unsuccessful treatment outcomes are essential.

- Strength of the study

As this retrospective study was conducted for the first time in this study area, the findings of this study will help healthcare professionals and policymakers to better understand the problems causing unsuccessful treatment outcomes. This study fills the gap of information regarding treatment outcomes and variables associated with unsuccessful outcomes.

- Limitations

A limitation of this study could be that since the data collected was retrospective secondary data and the data source (which was the standard TB register) did not capture detailed information, some cases were missing or had inaccurate data. Moreover, the data were collected from one site, Adare General Hospital, which might not be representative enough to provide a general conclusion, since patients from other facilities have different profiles.

- Biases

Potential biases could be associated with the dependence of this study on data from a registration book and analyses based on a single center.

## Figures and Tables

**Table 1:** Socio-demographic and disease-related characteristics of TB patients in Adare General Hospital, Southern Ethiopia.

Variable	Frequency	Percentage (%)
Sex		
Male	620	55.3
Female	502	44.7
Residence		
Urban	748	66.7
Rural	374	33.3
Age in years		
0–14	101	9
15–24	362	32.3
25–34	328	29.2
35–44	172	15.3
45–54	74	6.6
55–64	50	4.5
≥65	35	3.1
Microscopy profile of TB		
PTB+	319	28.4
PTB−	352	31.4
EPTB	452	40.2
Category of TB patients		
New cases	1064	94.8
Relapse cases	48	4.3
Treatment after failure	2	0.2
Transferred cases	8	0.7
Died	54	4.7
HIV test result		
Unknown	6	0.5
Positive	238	21.2
Negative	878	78.3

PTB+ = pulmonary tuberculosis-positive; PTB− = pulmonary tuberculosis-negative; EPTB = extra-pulmonary TB; HIV = human immunodeficiency virus.

**Table 2:** Tuberculosis treatment outcomes by demographic and profiles of TB patients in Adare General Hospital, Southern Ethiopia.

Variables		Successful		Unsuccessful			Total N (%)
		Cured N (%)	Completed N (%)	Died N (%)	Defaulter N (%)	Failure N (%)	
Sex	Male	172 (27.7)	401 (64.7)	28 (4.5)	18 (2.9)	1 (0.2)	620 (55.3)
	Female	112 (22.3)	352 (70.1)	25 (5)	11 (2.2)	2 (0.4)	502 (44.7)
Residence	Urban	183 (24.5)	524 (70.1)	27 (3.6)	13 (1.7)	1 (0.1)	748 (66.7)
	Rural	101 (27)	229 (61.2)	26 (7)	16 (4.3)	2 (0.5)	374 (33.3)
Age	0–14	9 (8.9)	88 (87.1)	1 (1)	2 (2)	1 (1)	101 (9)
	15–24	111 (30.7)	231 (63.8)	9 (2.4)	10 (2.8)	1 (0.3)	362 (32.3)
	25–34	98 (29.9)	211 (64.3)	14 (4.3)	5 (1.5)	0 (0.0)	328 (29.2)
	35–44	32 (18.6)	12 (70.3)	12 (7)	7 (4.1)	0 (0.0)	172 (15.3)
	45–54	19 (25.6)	45 (60.8)	8 (10.8)	1 (1.4)	1 (1.4)	74 (6.6)
	55–64	8 (16)	31 (62)	7 (14)	4 (8)	0 (0.0)	50 (4.5)
	≥65	7 (20)	26 (74.3)	2 (5.7)	0 (0.0)	0 (0.0)	35 (3.1)
Microscopy profile of TB	PTB+	278 (87.1)	12 (3.8)	15 (4.7)	13 (4.1)	1 (0.3)	319 (28.4)
	PTB−	4 (1.1)	324 (92.1)	20 (5.7)	4 (1.1)	0 (0.0)	352 (31.4)
	EPTB	2 (0.4)	417 (92.5)	18 (4)	12 (2.7)	2 (0.4)	451 (40.2)
HIV status	Unknown	0 (0.0)	6 (1)	0 (0.0)	0 (0.0)	0 (0.0)	6 (0.5)
	Positive	40 (16.8)	151 (63.4)	35 (15)	11 (4.6)	1 (0.4)	238 (21.2)
	Negative	244 (27.8)	596 (68)	18 (2)	18 (2)	2 (0.2)	878 (78.3)
Overall Total		284 (25.3)	753 (67.1)	53 (4.7)	29 (2.6)	3 (0.3)	1122 (100)

N = number of cases; % = percent of cases; PTB+ = pulmonary tuberculosis positive; PTB− = pulmonary tuberculosis negative; EPTB = extra-pulmonary TB; HIV = human immunodeficiency virus.

**Table 3:** Trend of treatment outcomes of all forms of registered TB cases in Adare General Hospital, Southern Ethiopia.

Treatment Outcome	Year					Total (%)
	2013	2014	2015	2016	2017	
Cured (%)	50 (20.8%)	97 (26.8%)	67 (23.6%)	57 (30%)	13 (33.3%)	284 (25.3)
Treatment completed (%)	183 (76.3%)	242 (66.9%)	197 (67.7%)	110 (57.8%)	21 (53.8%)	753 (67.1)
Successful						
Total (%)	233 (97%)	339 (93.6%)	264 (90.7%)	167 (87.9%)	34 (87.2%)	1037 (92.4)
Died (%)	6 (2.5%)	9 (2.5%)	19 (6.5%)	15 (7.9%)	4 (10.3%)	53 (4.7)
Defaulted (%)	1 (0.4%)	13 (3.6%)	6 (2.1%)	8 (4.2%)	1 (2.6%)	29 (2.6)
Treatment Failure (%)	0 (0.0)	1 (0.3%)	2 (0.7%)	0 (0.0)	0 (0.0)	3 (0.3)
Unsuccessful Total (%)	7 (2.9%)	23 (6.4%)	27 (9.3%)	23 (12.1%)	5 (12.8%)	85 (7.6)
Overall Total (%)	240 (21.4)	362 (32.3)	291 (25.9)	190 (16.9)	39 (3.5)	1122 (100)

**Table 4:** Factors associated with TB treatment outcome among TB patients in Adare General Hospital, Southern Ethiopia.

Variables	Total No.	Treatment Outcome		COR		AOR	
		Successful (%)	Unsuccessful (%)				
Residence							
Urban	748	707 (63)	41 (3.7)	2.29 (1.47, 3.58)	0.00	1.43 (0.27,0.67)	0.00
Rural	374	330 (29.4)	44 (3.9)	1		1	
HIV status							
Positive	238	191 (17)	47 (4.2)	1		1	
Negative	884	846 (75.4)	38 (3.4)	1.34 (0.11, 0.28)	0.00	5.47 (3.47, 8.63)	0.00
Types of TB							
PTB+	319	290 (25.8)	29 (2.6)	1		1	
PTB−	352	328 (29.3)	24 (2.1)	1.31 (0.78, 2.21)	0.11	0.95 (0.55, 1.65)	0.87
EPTB	451	419 (37.3)	32 (2.9)	0.96 (0.59,1.70)	0.18	0.70 (0.26, 1.86)	0.478

% = percent; COR = crude odds ratio; AOR = adjusted odd ratio; PTB+ = pulmonary tuberculosis-positive; PTB− = pulmonary tuberculosis-negative; EPTB = extra-pulmonary TB; HIV = human immunodeficiency virus.

## Data Availability

All the data generated and analyzed during the present study are included in this manuscript and can be accessed from the corresponding author.

## Abbreviations

AOR	Adjusted odds ratio
DOTS	Directly Observed Treatment Short-Course
EPTB	Extra-pulmonary TB
FMOH	Federal Ministry of Health
MTB	Mycobacterium tuberculosis
NTLCP	National TB and Leprosy Control Program
SNNPR	Southern Nations, Nationalities, and Peoples‘ Region
SPSS	Statistical Package for Social Science
PTB	Pulmonary tuberculosis
TB	Tuberculosis
WHO	World Health Organization
